# Molecular Characterization of Host-Specific Biofilm Formation in a Vertebrate Gut Symbiont

**DOI:** 10.1371/journal.pgen.1004057

**Published:** 2013-12-26

**Authors:** Steven A. Frese, Donald A. MacKenzie, Daniel A. Peterson, Robert Schmaltz, Teresa Fangman, You Zhou, Chaomei Zhang, Andrew K. Benson, Liz A. Cody, Francis Mulholland, Nathalie Juge, Jens Walter

**Affiliations:** 1 Department of Food Science and Technology, University of Nebraska, Lincoln, Nebraska, United States of America; 2 Institute of Food Research, Norwich Research Park, Norwich, United Kingdom; 3 Johns Hopkins University School of Medicine, Department of Pathology, Baltimore, Maryland, United States of America; 4 Center for Biotechnology, University of Nebraska, Lincoln, Nebraska, United States of America; The University of Texas Health Science Center at Houston, United States of America

## Abstract

Although vertebrates harbor bacterial communities in their gastrointestinal tract whose composition is host-specific, little is known about the mechanisms by which bacterial lineages become selected. The goal of this study was to characterize the ecological processes that mediate host-specificity of the vertebrate gut symbiont *Lactobacillus reuteri*, and to systematically identify the bacterial factors that are involved. Experiments with monoassociated mice revealed that the ability of *L. reuteri* to form epithelial biofilms in the mouse forestomach is strictly dependent on the strain's host origin. To unravel the molecular basis for this host-specific biofilm formation, we applied a combination of transcriptome analysis and comparative genomics and identified eleven genes of *L. reuteri* 100-23 that were predicted to play a role. We then determined expression and importance of these genes during *in vivo* biofilm formation in monoassociated mice. This analysis revealed that six of the genes were upregulated *in vivo*, and that genes encoding for proteins involved in epithelial adherence, specialized protein transport, cell aggregation, environmental sensing, and cell lysis contributed to biofilm formation. Inactivation of a serine-rich surface adhesin with a devoted transport system (the SecA2-SecY2 pathway) completely abrogated biofilm formation, indicating that initial adhesion represented the most significant step in biofilm formation, likely conferring host specificity. In summary, this study established that the epithelial selection of bacterial symbionts in the vertebrate gut can be both specific and highly efficient, resulting in biofilms that are exclusively formed by the coevolved strains, and it allowed insight into the bacterial effectors of this process.

## Author Summary

The bacterial communities found in the vertebrate gastrointestinal tract are remarkably stable and host-specific. However, the ecological and molecular processes that facilitate the selection of microbial symbionts and the exclusion of detrimental bacteria are not well understood. Here, we explore the mechanisms that underlie colonization and biofilm formation in specific strains of the gut symbiont *Lactobacillus reuteri*. When previously germ-free mice are colonized by individual strains of *L. reuteri*, only strains originating from rodents formed biofilms on the forestomach epithelium. Genomic, proteomic, and molecular analysis provide a detailed look into the host-specific molecular processes, such as adhesion, that contribute to colonization and biofilm formation. Our findings demonstrate high fidelity in the epithelial selection of a bacterial gut inhabitant, which can differentiate even between strains of the same species, strengthening the notion that some relationships between vertebrates and their microbial symbionts are highly coevolved and exclusive.

## Introduction

Most members of the animal kingdom form associations with symbiotic microorganisms that are often of fundamental importance for their biology [1]. These symbioses vary in terms of their effects on the host and the evolutionary and ecological processes that maintain the partnership. To date, host-microbial symbiosis is best understood in invertebrates such as insects, nematodes, and the Hawaiian squid *Euprymna scolopes* [2], [3], [4], [5], [6]. These symbioses are often mutualistic, coevolved, and remarkably specific, with the host being able to select for the correct symbiotic partners and stably maintain them over ecological and evolutionary time-scales [7]. Host specificity is considered one of the factors that support the evolution and maintenance of mutualistic interactions [8], and scientists have begun to use model systems to identify the molecular mechanisms by which exclusive symbiotic alliances become established [2].

Vertebrates also form relationships with microbial populations that play important roles in their biology and development, and therefore qualify as symbioses [9], [10]. Microbial communities associated with vertebrates are generally more diverse than those found with invertebrates, comprising hundreds of microbial species, most of which are bacteria. The densest bacterial population associated with vertebrates is found in the gastrointestinal (GI) tract (the gut microbiota), and as a whole, this community makes important contributions to the host in the form of nutrient provision, resistance to infections, and development of immune system functions [1], [10]. Despite the importance of the gut microbiota, there is still little known on how bacterial populations become acquired, are stably maintained by the host. 16S rRNA surveys revealed that the fecal microbiota of mammals is to a large degree specific for their particular host species [11], [12] and remarkably stable [13], [14], indicating that mechanisms are in place to recruit and maintain selected bacterial populations. However, in contrast to microbial symbioses in invertebrates, virtually nothing is known about the molecular processes by which recognition, selection, and capture of bacterial lineages are conferred in vertebrates.

*Lactobacillus reuteri* is a gut symbiont present in a variety of vertebrate species, likely benefiting its host [10]. We have recently demonstrated, by using a combination of population genetics and comparative genomics, that the species is composed of host-specific clades [15] with lineage-specific genomic differences that reflect the niche characteristics in the GI tract of respective hosts [16]. Experiments in *Lactobacillus*-free mice to measure the ecological fitness of strains originating from different hosts supported host adaptation, as only rodent strains colonized mice efficiently [16]. Overall, the findings indicated that *L. reuteri* is a host specific symbiont, and the separate lineages within the species suggest that host restriction was maintained over evolutionary time spans, allowing host-driven diversification [15], [16]. We have demonstrated the ecological significance of a subset of rodent-specific *L. reuteri* genes in the context of the murine gut [16], but the mechanisms by which these genes influence host colonization and the molecular processes that mediate host specificity have not been systematically investigated.

In rodents, pigs, chickens, and horses, lactobacilli form large populations in proximal regions of the GI tract, and they adhere directly to the stratified squamous epithelium present at these sites [17], [18]. In mice and rats, adherence occurs in the forestomach [19], and this process appears to be important with regards to the ecological fitness of the bacteria [20]. The epithelial associations formed can be considered biofilms as the bacteria are arranged in multiple layers and are encased in a polysaccharide matrix [21], [22]. Although there is ample microscopic evidence that supports the existence of these biofilms [17], [23], [24], there is very limited information on how they form and the underlying molecular processes. In addition, while studies have shown that the adherence of *Lactobacillus* isolates to epithelia and epithelial cells is host-specific [19], [25], [26], it has not been established if differences in biofilm formation contribute to the host specificity observed within the species *L. reuteri*.

In this study, we used experiments with monoassociated mice and demonstrated that epithelial biofilm formation in *L. reuteri* is dependent on host origin of the stains. To gain insight into the molecular basis of host-specific biofilms, we identified genes of *L. reuteri* 100-23 that are upregulated during growth in an *in vitro* biofilm, are lineage-specific and are predicted to contribute to biofilm formation, or are orthologs of genes from other bacteria with established roles in biofilm formation. The importance of these genes to *in vivo* biofilm formation was then determined by monitoring their expression level and assessing the phenotype of null mutations in mouse colonization experiments.

## Results

### Temporal characterization of *L. reuteri* biofilms in the forestomach of mice

**Figure 1:**
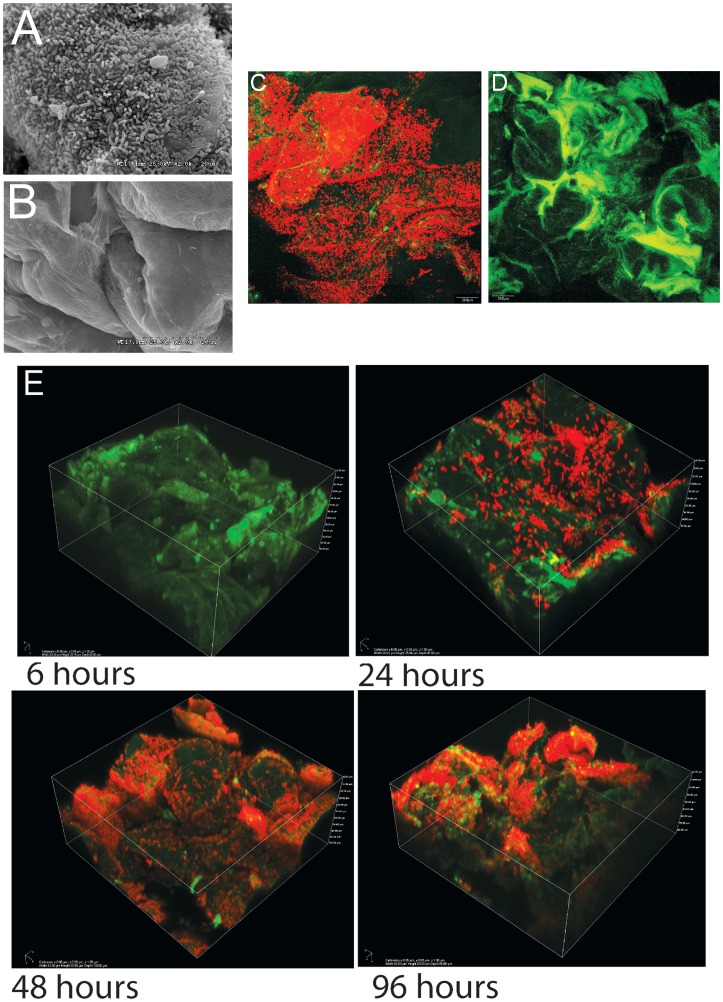
Biofilms of *Lactobacillus reuteri* 100-23 on the keratinized squamous stratified epithelium of the mouse forestomach. (A) Scanning electron microscopy micrograph of forestomach epithelium of ex germ-free mice two days after a single gavage of 10^7^ CFU *L. reuteri* 100-23. (B) Same epithelium in a germ-free mouse. (C) Image of biofilm after two days of colonization obtained with confocal microscopy after staining with propidium iodide (bacterial cells stain red). (D) Same tissue obtained from a germ-free mouse visualized by confocal microscopy. (E) Confocal 3D images showing the mouse forestomach epithelium after 6, 24, 48, and 96 h of inoculation.

As shown previously in ex-Lactobacillus-free BALB/c mice [21], [22], *L. reuteri* 100-23 forms dense layers of cells on the forestomach epithelium of monoassociated (ex-GF) Swiss Webster mice that can be visualized by both scanning electron microscopy (SEM) and confocal microscopy (Figure 1A–D). A subset of the bacterial cells were directly attached to the epithelium and protruding epithelial cells, while other bacteria were attached to bacteria, forming multiple layers of cells (**Video S1**). Temporal characterization of colonization revealed that it required 48 hours for a mature biofilm to develop (Figure 1E). After 24 hours, individual cells were found adhering directly to the epithelium, and microcolonies, composed of clumps of cells, became visible. 48 hours after gavage, luminal bacterial populations in the stomach reached a stable plateau of around 10^8^ cells/gram (data not shown), and the biofilm appeared to reach a final density as no further increase occurred. However, even within mature biofilms, colonization was patchy, with some areas being densely populated by various layers of cells, while others showed few adherent cells. These patterns are likely caused by the continuous shedding of epithelial cells, resulting in vacant areas that have to be recolonized.

### *L. reuteri* biofilm formation is strictly dependent on the strain's host origin

We developed an experimental approach by which to compare *in vivo* biofilm formation of *L. reuteri* strains after 48 hours of colonization in germ-free mice. Although our previous studies were in *Lactobacillus*-free mice which approximate a microbiota functionally equivalent to conventional animals [16], we specifically chose monoassociated mice here as they allow the specific study of biofilms and the underlying bacterial factors in the absence of competitive interactions. Competitive interactions that affect colonization unrelated to biofilms (e.g. interference through bacteriocins, competition for substrates, bacteriophages) would confound our ability to first interpret the exact ecological role of biofilms, and second, to identify bacterial factors that specifically contribute to their formation. In our case, the monoassociated mouse model was necessary to exclusively compare biofilm formation of rodent and non-rodent strains as the latter are poor colonizers of mice with a competitive microbiota [16].

**Figure 2:**
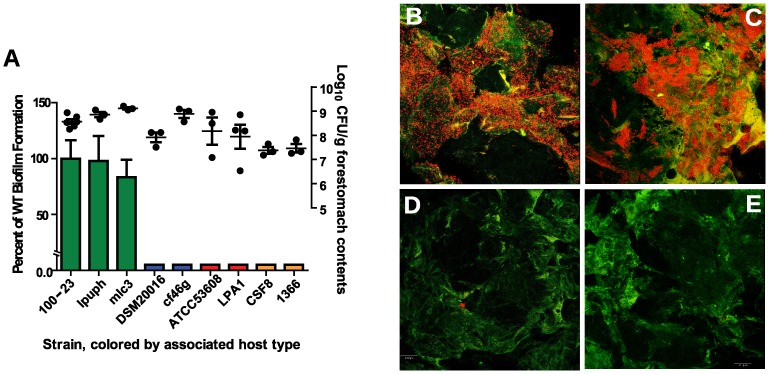
Biofilm formation of *L. reuteri* strains is host specific. (A) Quantification of biofilm density (relative to biofilm of strain 100-23) by confocal microscopy and cell counts in forestomach contents of *L. reuteri* strains two days after gavage with a single dose of ∼10^7^ cells. Bars are color coded according to host origin (green, rodent; blue, human; red, pig, and orange, chicken). Confocal micrographs showing density and pattern of bacteria (red) by strains (B) Lpuph (mouse), (C) Mlc3 (mouse), (D) DSM20016^T^ (human), and (E) ATCC 53608 (pig).

Using this mouse model, biofilm formation of nine wild-type strains (Table 1) originating from different hosts (mouse, rat, human, pig, and chicken) was evaluated. Biofilms were quantified by confocal microscopy, measuring the pixel area in images where bacterial cells were stained with propidium iodide. The analysis revealed adherence of rodent strains to the forestomach epithelium and biofilm formation (Figure 2A, B, and C), while non-rodent strains were virtually absent from the epithelium (Figure 2A, D, and E). Interestingly, in the absence of competition, both rodent and non-rodent strains reached similar luminal populations (10^7^ to 10^9^ CFU/gram)(Figure 2A).

**Table 1:** Strains used in this study.

Strain	Relevant Characteristics	Source or Reference
L. reuteri 100-23	Rat gastrointestinal isolate	[26]
*L. reuteri* 100-23C	Plasmid-cured derivative of strain 100-23	[53]
*L. reuteri* 100-23C *cgl* mutant	Cystathionine γ-lyase inactivated	This study
*L. reuteri* 100-23C *ureC* mutant	Urease α-subunit inactivated	This study
*L. reuteri* 100-23C *lrgA* mutant	*lrgA* inactivated	This study
*L. reuteri* 100-23C *lytS* mutant	*lytS* inactivated	This study
*L. reuteri* 100-23C *lysM2* mutant	LysM-domain protein inactivated	This study
*L. reuteri* 100-23C *lysM3* mutant	LysM-domain protein inactivated	This study
*L. reuteri* 100-23C *70430* mutant	Two-component system inactivated	[16]
*L. reuteri* 100-23C *secA2* mutant	SecA2-transport system inactivated	[16]
*L. reuteri* 100-23C *lr70902* mutant	Large surface protein inactivated	[16]
*L. reuteri* 100-23C *lr70532* mutant	Two-component system inactivated	[16]
*L. reuteri* 100-23C *lsp* mutant	Large surface protein inactivated	[30]
*L. reuteri* Lpuph	Mouse isolate	[16]
*L. reuteri* Mlc3	Mouse isolate	[16]
*L. reuteri* DSM20016^T^ (ATCC 23272)	Human isolate	Type strain
*L. reuteri* cf4-6g	Human isolate	[15]
*L. reuteri* ATCC 53608	Pig isolate	[54]
*L. reuteri* LPA1	Pig isolate	[15]
*L. reuteri* CS-F8	Chicken isolate	[55]
*L. reuteri* 1366	Chicken isolate	[15]

### Identification of *L. reuteri* genes predicted to be involved in biofilm formation

To gain insight into the molecular processes that facilitate host-specific biofilm formation, we identified genes of *L. reuteri* 100-23 predicted to play a role, using three independent approaches of gene discovery. First, we performed a transcriptome analysis to identify genes whose expression is upregulated during *in vitro* biofilm growth. Second, we identified genes specific to host-confined *L. reuteri* lineages [16] that were also predicted to be involved in biofilm formation. Finally, we searched for genes that were not host-specific but were orthologs of genes with established roles in bacterial biofilms.

#### Transcriptome analysis of *L. reuteri* during *in vitro* biofilm growth

Genes of *L. reuteri* 100-23 that were differentially expressed during biofilm formation in a flow cell when compared to regular batch culture were identified by microarray analysis and whole transcriptome sequencing (RNA-seq). Microarray analysis identified 91 genes upregulated more than twofold during growth in the biofilm; an additional 37 genes showed greater than twofold overexpression during planktonic growth (Table S1). Amongst the loci with the most significant upregulation during biofilm growth were a cystathionine gamma-lyase gene cluster, four genes encoding for putative surface proteins containing LysM domains/YG motifs, and genes encoding the LrgAB system (which is involved in the control of cell death and lysis in *Staphylococcus aureus* biofilms [27]). RNA-seq confirmed most of the findings obtained with the microarray analysis (Table S1), but also revealed that genes within the urease gene cluster were upregulated in the biofilm, which were not detected by the microarray.

#### Lineage-specific genes predicted to be involved in biofilm formation

Our previous comparative genomic analysis identified several host-specific genes that contributed to ecological fitness of *L. reuteri* 100-23 in *Lactobacillus*-free mice, and that were annotated to have a putative role in biofilm formation [16]. Rodent and porcine *L. reuteri* strains possess an accessory Sec system (the SecA2-SecY2 system), which is present in a small number of Gram-positive species and is typically specialized in the transport of heavily glycosylated adhesins [28]. In order to determine the spectrum of proteins of *L. reuteri* 100-23 secreted by the SecA2-SecY2 pathway, we performed a proteomic analysis and compared extracellular and cell wall-associated proteins in the *secA2* mutant [16] and the wild-type 100-23C strain. In the wild-type strain, 16 proteins were identified with high confidence to be secreted or associated with the cell surface (Table S2). Many of these proteins were predicted to be cell wall-associated, possessing cell wall anchors or putative cell wall binding motifs. Interestingly, the only detectable difference between the *secA2* mutant and the wild-type was the absence of the surface protein Lr70902 in the spent media from the *secA2* mutant, and a significant reduction of this protein in the cell surface extract (Table S2). These findings suggested that Lr70902, whose gene is colocalized with the *secA2* gene cluster [16], is the primary cell-wall associated protein of strain 100-23 that is secreted through the accessory SecA2 pathway during *in vitro* growth. The presence of a few cytoplasmic proteins detected at low levels in the extracellular and cell surface extracts from both strains (data not shown) suggested that the residual amount of Lr70902 in the *secA2* mutant's cell wall may be due to low levels of cell lysis, although residual transport through other secretory pathways cannot be ruled out.

Lr70902 (2180 aa) shows sequence homology with bacterial adhesins, possesses a LPXTG cell wall anchor, and it displays characteristics of a protein secreted through the SecA2 system [28]. Like other SecA2-secreted proteins, it is extremely serine rich and contains an unusually long signal peptide. The serine-rich motif ‘SVSMSESLSN’ is repeated identically and in succession 74 times in Lr70902, and a similar repeating pattern of serine residues is also found in the SecA2-secreted fimbrial adhesin (Fap1) of *Streptococcus parasanguinis* [29].

The comparative genomic analysis further identified two separate two-component regulatory systems (TCS) as being host specific, and these systems might contribute to quorum sensing or other regulatory functions during biofilm formation [16]. One of these systems, comprised of a histidine kinase (Lr70430), a LytR/AlgR family response regulator (Lr70431), and a bacteriocin processing peptidase (Lr70432), was only found in strain *L. reuteri* 100-23 [16]. The other system was more conserved among rodent strains [16] and contains a putative histidine kinase (Lr70529), a response regulator of the LytR/AlgR family (Lr70530), a bacteriocin-like peptide (Lr70531), an ABC-type bacteriocin transporter (Lr70532), and an ABC-type bacteriocin/lantibiotic exporter, containing an N-terminal double-glycine peptidase domain (Lr70533).

#### Orthologous genes with established roles in bacterial biofilm formation

*L. reuteri* genomes contain several orthologues of bacterial genes with demonstrated roles in biofilm formation. All *L. reuteri* strains with the exception of those in lineage VI possess a LytS/LytR system, which is a TCS that serves as a regulator of cell autolysis in *S. aureus*, contributing to biofilm formation by generating a DNA matrix within the biofilm [27]. The antiholin LrgA is one of the genes regulated by LytS/LytR in *S. aureus*. Like in *S. aureus*, the LytS/LytR system of *L. reuteri* 100-23 (Lr69269/Lr69270) is found directly upstream of the *lrgAB* operon (Lr69271/Lr69272) (Figure S1), which was upregulated during *in vitro* biofilm formation of *L. reuteri* 100-23 (see above).

*L. reuteri* strains possess several surface proteins that are predicted to be involved in biofilm formation or epithelial adherence, including the Lsp protein from *L. reuteri* 100-23. Lsp is a homologue of Esp and Bap from *Enterococcus faecalis* and *S. aureus*, which are proteins involved in biofilm formation [30]. Previous work showed that Lsp contributes to ecological performance of *L. reuteri* 100-23C in the mouse gut, and *ex vivo* adherence assays suggested that this was through its role in initiating adherence to the epithelium [30].

### Functional characterization of genes predicted to be involved in biofilm formation

The information obtained from the combined gene discovery approach was used to select eleven genes of *L. reuteri* 100-23 for functional studies (Table 2). Due to the high number of differentially regulated genes during *in vitro* biofilm formation, not all of the genes identified as over-expressed could be tested in mouse experiments. Three of the four LysM/YG proteins whose expression was induced during *in vitro* biofilm growth (Lr69719, Lr69721, Lr71416) possess very similar LysM and YG domains with high homology (>80% amino acid homology) (Figure S2). Therefore, two of the genes (*lr70152* and *lr71416*), which typified this group of proteins in strain 100-23, were selected for further characterization.

**Table 2:** Genes of *L. reuteri* 100-23 selected for functional characterization.

Gene	Protein	Description	Putative Function	Reason for Study
***lr71416***	LysM2	LysM/YG Domain Protein	Aggregation; Described in *L. johnsonii*, *L. gasseri*, *L. acidophilus*	Upregulated in Biofilm
***lr70152***	LysM3	LysM/YG Domain Protein	Aggregation; Described in *L. johnsonii*, *L. gasseri*, *L. acidophilus*	Upregulated in Biofilm
***lr69271***	LrgA	LrgA biofilm regulator	Regulator of Biofilm formation; Described in *Staphylococcus aureus*	Upregulated in Biofilm
***lr69360***	Cgl	Cystathionine gamma lyase	Reactive Oxygen (RO) Resistance; Described in *L. reuteri* BR11	Upregulated in Biofilm
***lr70892***	SecA2	secA2 protein translocases	Transport of surface proteins to cell surface; Described in *Streptococcus gordonii*, *L. reuteri* 100-23	Host specific and predicted to secrete proteins related to biofilm formation and adhesion [28]
***lr70902***	Fap1-like protein	Serine-rich large surface protein	Adhesion to forestomach epithelium; described in *L. reuteri* 100-23	Host specific and predicted to be involved in adhesion [16]
***lr70532***		Putative ABC bacteriocin transporter	Quorum sensing	Host specific, and quorum sensing is often important for biofilm formation
***lr70430***		Histidine kinase of two-component regulatory system	Strain-specific regulatory system	Critical for ecological success [16]
***lr70114***	UreC	Urease enzyme, alpha subunit	Acid resistance	Host specific, and acid resistance has been shown to be important in biofilms [16]
***lr69269***	LytS	LytS regulator	Regulator of cell lysis during biofilm formation	Biofilm regulatory gene in *Staphylococcus aureus* [39]
***lr70580***	Lsp	Large surface protein	Putative adhesin	Homologe of biofilm related proteins and critical for ecological success [30]

#### Temporal examination of *in vivo* gene expression during colonization of the forestomach epithelium

Mice were monoassociated with *L. reuteri* 100-23, and the expression of the selected genes in cells adherent to the forestomach epithelium was determined by qRT-PCR at different time points (6 to 96 hours), encompassing the different steps of biofilm formation (Figure 1E). This analysis showed a wide spectrum of expression levels among the genes (Table 3). The *ureC* and *lsp* genes were upregulated more than 100-fold *in vivo* when compared to *in vitro* growth, while genes such as *lrgA*, *secA2*, *lr70902*, *lytS* and *lysM3* showed increases ranging from 5 to 30-fold. For most genes, expression was not temporarily regulated during *in vivo* biofilm formation, as no difference was detected between time points at 6, 12, and 24 hours (adherent cells and microcolonies) and time points >24 hours (mature biofilm). The only exception was the *lytS* gene (Lr69269), expression of which showed a progressive increase, reaching the highest level of expression after 96 hours of colonization.

**Table 3:** Gene expression fold change (SEM) during colonization of the forestomach epithelium, compared to batch culture as determined by qRT-PCR.

Gene	6 hrs	12 hrs	24 hrs	48 hrs	72 hrs	96 hrs
***secA2*** ** (Lr70892)** 1	17.37 (2.84)	25.01 (9.14)	2.45 (1.08)	11.99 (7.5)	58.30 (16.4)	14.74 (1.33)
***lr70902***	17.89 (0.78)	23.84 (10.4)	21.21 (7.15)	6.90 (3.14)	20.88 (4.83)	29.86 (15.3)
***lytS*** ** (Lr69269)** 2	5.82 (0.035)^a^	12.05 (5.56)^a^	6.20 (3.26)^a^	19.41 (0.12)^a, b^	22.13 (3.51)^a, b^	55.98 (17.9)^b^
***lysM3*** ** (Lr70152)**	4.58 (1.51)	5.66 (0.68)	0.43 (0.18)	13.43 (3.62)	6.89 (0.55)	5.06 (0.20)
***lysM2*** ** (Lr71416)**	0.14 (0.06)	0.25 (0.03)	0.06 (0.03)	0.13 (0.03)	0.22 (0.4)	0.15 (0.01)
***lr70532***	0.0078 (0.01)	0.77 (0.48)	2.07 (1.55)	2.84 (2.68)	0.18 (0.05)	0.18 (0.03)
*ureC* (Lr70114)	131.94 (43.5)	110.17 (31.1)	106.59 (62.7)	0.799 (0.43)	88.90 (23.9)	103.13 (7.52)
*lsp* (Lr70580)	96.79 (21.8)	64.80 (3.34)	0.80 (0.67)	35.86 (22.6)	71.65 (41.4)	70.86 (7.64)
*lr70430*	2.18 (1.24)	1.90 (0.70)	2.56 (2.10)	14.98 (13.6)	0.57 (0.06)	0.77 (0.33)
*cgl* (Lr69360)	2.17 (0.94)	4.24 (2.20)	1.65 (0.88)	2.12 (1.28)	4.94 (0.82)	1.89 (1.32)
*lrgA*(Lr69271)	7.33 (0.42)	6.53 (3.13)	2.85 (0.16)	4.23 (1.40)	6.59 (0.65)	4.38 (1.55)

^1^Genes in bold contribute to biofilm formation.

^2^Significant changes over time are shown by superscript groups (a,b).

#### Evaluation of the genes' contribution to *in vivo* biofilm formation

**Figure 3:**
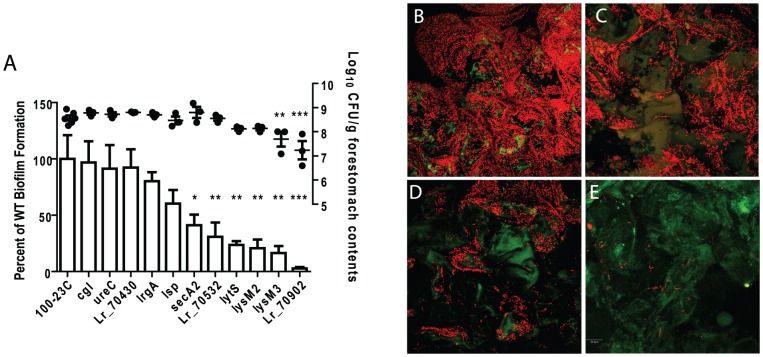
Characterization of *in vivo* biofilms of mutant strains of *L. reuteri* 100-23C. (A) Quantification of biofilm density (relative to biofilm of wild-type strain 100-23C) by confocal microscopy and cell counts in forestomach contents of *L. reuteri* mutants two days after gavage with a single dose of ∼10^7^ cells. ANOVA with Dunnett's multiple comparison test, *, *p*<0.05; **, *p*<0.01; ***, *p*<0.001). Confocal micrographs of forestomach tissue from mice colonized for two days with (B) wild type, (C) *secA2* mutant, (D) *lysM3* mutant, and (E) *lr70902* mutant.

To determine the importance of genes for *in vivo* biofilm formation, groups of germ-free mice (n = 3) were colonized for two days by mutant strains or wild-type 100-23C, and biofilm density was compared by confocal microscopy. These experiments revealed that several gene products were critical for biofilm formation (Figure 3). SecA2, the ABC-type bacteriocin transporter (Lr70532), LytS (Lr69269), and the two LysM-domain proteins (Lr71416 and Lr70152), showed a significant contribution to biofilm density, while mutation of *lr70902* almost completely eliminated epithelial associations (Figure 3E). Although these mutants were impaired in biofilm formation, they reached population densities in the forestomach lumen of >10^7^ cells/gram after two days of colonization (Figure 3A), indicating that genes are specifically involved in biofilm formation.

The inactivation of the other loci did not result in a significant reduction in *in vivo* biofilm formation. Interestingly, three of these loci (*lsp*, *ureC*, and *lr70430*) have been shown to contribute to ecological performance of *L. reuteri* 100-23C in the mouse gut ([16], [30] and unpublished observations). These genes may contribute either to functions not related to biofilm formation (e.g. acid resistance for the urease cluster), or they may be functionally redundant. For example, the *lsp* mutant showed a modest but not significant reduction in biofilm formation (Figure 3A). In this respect, it is relevant to point out that *L. reuteri* 100-23 possesses several paralogues of Lsp that could account for the residual function in the mutant. This concurs with previous findings in *Lactobacillus*-free mice which showed that the *lsp* mutant, although impaired *in vivo*, could still attach to the forestomach epithelium [30].

## Discussion

The vertebrate gut microbiota makes critical contributions to the host, but relationships have to be recapitulated each generation as vertebrates are germ-free at birth. Here we demonstrate that host epithelial selection of a bacterial symbiont can be highly specific in the mouse gut, allowing efficient differentiation between strains of the same species.

### Epithelial associations of *L. reuteri* qualify as biofilms and are host specific

Biofilms are populations of microorganisms that are concentrated at an interface (usually solid-liquid) and typically surrounded by an extracellular polymeric substance matrix, such as exopolysaccharides (EPS) [31]. Growth in epithelial biofilms can increase bacterial persistence in flowing habitats, and biofilms have therefore often been postulated to constitute an important hallmark of bacterial colonization of the intestinal tract [32]. However, evidence for the existence of biofilms in the intestinal tract is inconclusive [33], and findings by the Hansson laboratory indicated that most of the intestinal lining is covered by two layers of mucus that prevent direct contact of bacteria with the epithelium [34]. Bacteria are associated with the outer mucus layer, but this matrix remains loosely attached and is constantly replaced, and it is questionable if it would permit the formation of biofilms. In contrast, the forestomach epithelium in mice is not covered by mucus, allowing direct attachment of bacteria to the epithelial cells (Figure 1A). The temporal analysis of *L. reuteri* colonization of the forestomach epithelium using confocal microscopy extended our previous studies [21], [22] and revealed some of the classic features of biofilm formation, such as attachment followed by the formation of microcolonies (Figure 1E).

Most importantly, the ability to form biofilms on the forestomach epithelium is completely congruent with the host origin of the strains, with only rodent strains forming biofilms. It is important to point out that, in contrast to the findings in *Lactobacillus*-free mice [16], non-rodent strains were able to colonize germ-free mice (Figure 2A). However, even the presence of high numbers of bacteria in the lumen (10^7^ to 10^9^ CFU/gram) did not lead to attachment to the epithelium (Figure 2D and E), showing that epithelial capture is highly selective.

### Molecular processes that underlie *L. reuteri* biofilm formation

**Figure 4:**
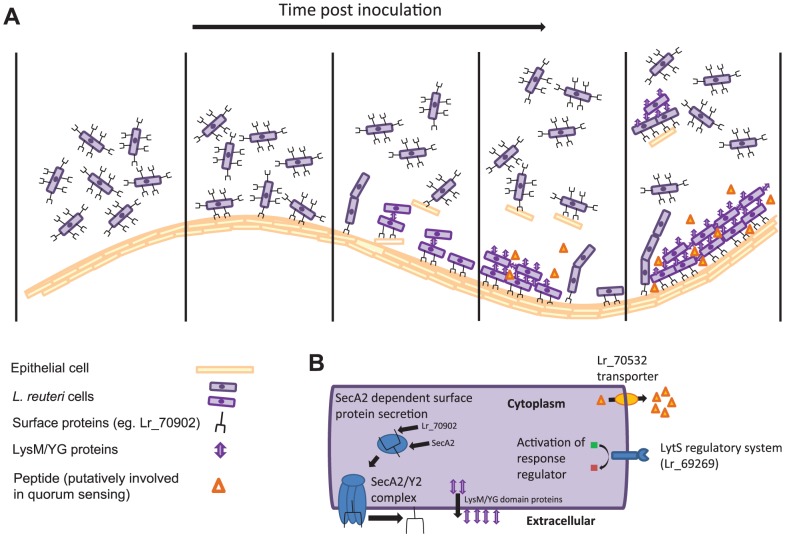
Model depicting the *in vivo* biofilm formation of *L. reuteri*. (A) Schematic summary illustrating steps of biofilm formation. (B) *L. reuteri* cells with the bacterial factors involved in biofilm formation.

The combination of transcriptomics and comparative genomics proofed a highly successful approach to identify biofilm-related genes, as mutation of six out of the eleven selected genes had a measurable effect. The information gained from the *in vivo* characterization of these genes allow inferences of the molecular processes that underlie *L. reuteri* biofilm formation in the mouse GI tract, and a preliminary model of the process is presented in Figure 4.

As a first step, individual *L. reuteri* cells adhere to the forestomach epithelium. The Fap1-like protein (Lr70902) is clearly of key importance for initial adherence, as the loss of this protein prevented almost all surface attachment (Figure 3E). Interestingly, our proteomic analysis suggested that *L. reuteri* 100-23 devotes a specialized secretion system (the SecA2 system) to Lr70902, underscoring the importance of this protein. The impairment of the *secA2* mutant in biofilm formation is therefore most likely caused by the reduction in Lr70902 transport to the cell surface. After initial attachment, *L. reuteri* biofilm development proceeds with the formation of micro and macrocolonies composed of cell aggregates (Figure 1E). The LysM/YG proteins of *L. reuteri* show characteristics of proteins that can induce aggregation in lactobacilli [35], [36], possibly by the N-terminal LysM domain binding to peptidoglycan [37] and the C-terminal YG-motif to carbohydrate moieties [36]. The contribution of these proteins to the formation of *in vivo* biofilms suggests an important role of autoaggregation in the overall process.

In several model organisms, biofilm formation is a carefully regulated process, which relies on cues from the population of cells (quorum sensing) and the environment [38]. Our experiments revealed two regulatory systems to contribute to *L. reuteri* biofilm formation (Figure 3); the LytS system and the TCS associated with an ABC bacteriocin transporter (Lr70532). During colonization, the expression of the *lyt*S gene increases progressively (Table 3), suggesting that it is induced in mature biofilms *in vivo*. In *S. aureus*, the LytSR system regulates expression of the *lrgAB* and *cidABC* operons, influencing cell lysis and the release of extracellular DNA (eDNA), which serves as a matrix in biofilms [27], [39]. It is not yet known how the LytSR system functions in *L. reuteri* and if eDNA plays a role in biofilms of the species. Likewise, the TCS associated with Lr70532, which might be involved in quorum sensing, is also clearly important for biofilm formation (Figure 3), yet its function and the genetic targets remain to be identified.

### Adhesion is likely the major host specific step in biofilm formation

While the ability to form biofilms in the rodent forestomach is a specific trait of rodent *L. reuteri* strains, most genes identified to contribute to biofilm formation are not unique to the rodent lineages of the species. The LysM/YG proteins and the LytS/R system are present in most *L. reuteri* genomes and are also found in related species (Figures S1 and S2). These findings suggest that biofilm formation may be an ancestral trait of the *L. reuteri* species, and accordingly, the species is a component of biofilms in the gut of rodents, pigs, and poultry. The complete absence of biofilm formation for the *lr70902* mutant suggests that it is the adhesion step that confers host specificity. Homologues of Lr70902 are only found in rodent and pig isolates of *L. reuteri* (in which they are always co-localized with the SecA2 gene cluster), and these proteins may fulfill a keystone role in specifically binding to the epithelium in their respective hosts. The low sequence similarity between the proteins of rodent and pig strains might account for the observed host specificity, but experiments are needed to test this hypothesis.

### Implications of host-specific biofilms for the ecology and evolution of *L. reuteri*

Several of the genes identified during this study as important in *in vivo* biofilm formation (*secA2*, *lr70902*, *lr70532*) were previously shown to strongly contribute to the ecological performance of *L. reuteri* in *Lactobacillus*-free mice [16], indicating that biofilm formation represents the key ecological process for gut colonization and likely the main mechanism by which host specificity is conferred [16]. Thus, differences in the ability to form biofilm provide an explanation for why rodent strains outcompete non-rodent strains in the gut of mice [15], and why non-rodent strains fail to efficiently colonize *Lactobacillus*-free mice [16]. In addition, the impaired ecological performance of the *secA2*, *lr70902*, *lr70532* mutants in *Lactobacillus*-free mice indicates that observations made in monoassociated mice are also relevant in a more complex setting, and that the gene functions remain relevant when a bacterial community is present.

From an evolutionary perspective, it is important to point out that the biofilm phenotypes of *L. reuteri* strains are completely consistent with the inferred phylogeny of the species [15]; non-rodent strains, which cluster separately from rodent strains, do not form biofilms, while isolates from both mice and rats form biofilms and cluster together in phylogenetic clades. This coherence suggests that host-specific biofilms are the result of a long-term evolutionary process, and the high fidelity of the epithelial selection provides a mechanism by which *L. reuteri* could diversify into host-specific lineages [15], [16].

### Is epithelial selection a common mechanism for symbiont capture in vertebrates?

As described above, mammals are able to select a host specific gut microbiota, but most microbes that reside in the intestinal tract are unlikely to maintain direct associations with the epithelium due to the presence of mucus [34]. It is not yet known whether these associations are sufficient to maintain stable symbiotic relationships over ecological and evolutionary time spans. However, most human isolates of *L. reuteri* show adherence to mucus, while rodent isolates do not [40], and this phenotype might be an adaptation to the human gut, which lacks a mucus-free stratified epithelium. In addition, computer modeling revealed that epithelial selection could be achieved through specific secretions provided by the host (e.g. nutrients such as glycoconjugates) [41]. Accordingly, it has been shown that *Bacteroides fragilis* is stably established in the colonic crypts, probably by being able to utilize a specific glycan structure provided by the host [42]. Nutrient-based epithelial selection is predicted to overrule competitive disparities between microbes, even those that result from large differences in growth rates [41]. This process could be highly relevant, as it would allow the host to select for true mutualists that bear fitness disadvantages due to the provision of costly benefits. Thus, epithelial selection, whether mediated through direct adhesion, as shown for *L. reuteri*, or through secretion, provides a mechanism for the selection of beneficial microbial populations in the vertebrate gut and a stabilization of mutualism.

### Conclusion

As a reservoir of potential pathogens, the gut microbiota has the ability to harm the host, especially if perturbed. In addition, evolutionary theory predicts that characteristics of the vertebrate microbiota, such as genetic diversity and horizontal transmission, create opportunities for conflict that can destabilize mutualistic partnerships [43]. It is therefore important for the host to not only have the capability to select beneficial microbes at every new generation, but also to stably maintain them over longer timescales to align fitness interests between the host and the symbiont [8]. The work here has contributed novel insight into the characteristics of the microbial symbiosis in the vertebrate GI tract in that it demonstrated highly efficient epithelial differentiation of bacterial strains, providing a mechanism for fidelity during transmission. The findings suggest that some *L. reuteri*-host interactions utilize similar mechanisms as described for invertebrate symbiosis (specific adherence, biofilms, cell aggregation) and pathogen-host interactions (SecA2, LytSR), but more work is necessary to elucidate the exact role of these bacterial factors in vertebrate host colonization in the context of beneficial alliances. Most importantly, the findings suggest that microbial symbiosis in vertebrates can display a high level of host specificity, suggesting that it might be more coevolved, exclusive, and obligate than so far recognized.

## Methods

### Ethic statement

All mouse experiments were performed with approval of the Institutional Animal Care and Use Committee of the University of Nebraska (Project ID 731).

### Strains and media used in the study

Strains used in this study are described in Table 1. The genetic work was performed with a plasmid-free variant of *Lactobacillus reuteri* 100-23, a rat isolate that belongs to the rodent-specific lineage III of the species [15]. The genome sequence for this organism has been determined (Genbank accession number: NZ AAPZ00000000.2). This strain has also been used in previous experiments examining biofilm formation *in vivo* in the rodent host [21], [22]. Bacteria were cultured anaerobically on modified MRS (mMRS) medium (MRS supplemented with 10 g/L maltose and 5 g/L fructose) at 37°C, unless otherwise noted. Inocula for mouse experiments were prepared by growing *L. reuteri* strains for 14 hours in liquid culture before recovering the cells by centrifugation (4000× RPM for 10 minutes). Prior to gavage, *L. reuteri* cells were washed twice with phosphate-buffered saline (PBS, pH 7.0) and suspended in the same buffer to generate the inocula.

### Mouse experiments

Germ-free Swiss Webster mice were maintained at the University of Nebraska Gnotobiotic Mouse Facility. For experiments to compare *in vivo* biofilm formation among strains, germ-free mice (6–16 weeks of age) were moved to sterile, individually ventilated biocontainment cages (Allentown Inc, Allentown, NJ, USA). Mice in a treatment group (n = 3) were housed together, and each mouse was gavaged with 100 µL of a cell suspension containing 10^7^ viable cells of *L. reuteri*. After 48 hours of colonization, mice were sacrificed by CO_2_ asphyxiation and the stomachs were obtained, contents were removed, and the forestomachs were fixed for microscopy. Bacterial numbers were determined in forestomach and/or cecal contents by plate count on mMRS. Each experiment included a sterile control group, where 1 or 2 mice were gavaged with sterile PBS instead of *L. reuteri*. Forestomach contents were cultured anaerobically on Brain Heart Infusion (BHI) Agar and mMRS to confirm the sterility of the ventilator system and the mouse cohort. In addition, from each cage of *L. reuteri* colonized mice, contents from one forestomach and one cecum were also cultured anaerobically on BHI Agar to control for bacterial growth other than *L. reuteri* (BHI does not support the growth of *L. reuteri* but is a commonly used universal medium, and is therefore suitable to detect potential contaminants). The mice in ventilated biocontainment cages remained germ-free over the duration of the experiments, as no biofilms were detected in mice that received PBS, and no growth occurred in any of the mice on BHI agar (data not shown).

For time course colonization experiments with *L. reuteri* 100-23 (Figure 1E and Table 3), eighteen 6–9 week old germ-free Swiss Webster mice were housed in three cages in a germ-free isolator and gavaged with 10^7^ CFU of the organism. One mouse per cage was removed 6, 12, 24, 48, 72, and 96 hours after gavage. Mice were sacrificed at indicated timepoints by CO_2_ asphyxiation, and tissue was immediately transferred to fixatives for microscopy, or transferred to RNase-free bead beating tubes and snap frozen in liquid nitrogen for RNA extraction (see below).

### Investigation of *in vivo* biofilms by SEM

Forestomach tissues were fixed in 0.1 M Sorenson's phosphate buffer containing 2.5% EM grade glutaraldehyde (Electron Microscopy Sciences, Hatfield, PA USA) and stored at 4°C until use. Fixed tissues were critical point dried and palladium/gold-sputter coated, and samples were visualized using a Hitachi S3000N scanning electron microscope (Hitachi High Technologies America, Schaumburg, Illinois).

### Visualization and quantification of in vivo biofilm formation by confocal microscopy

Forestomach tissues were fixed immediately in 3% formalin/phosphate-buffered saline (PBS, pH 7.0) for 30 min and then transferred to fresh 3% formalin/PBS pH 7.0 and stored at 4°C until usage. Samples were transferred to PBS pH 7.0 to remove residual methanol, and maintained for 60 min with one exchange of buffer after 30 min. Tissues were stained in 5 µg/mL propidium iodide (in PBS, pH 7.0) for 10 min. Samples were washed twice in PBS (pH 7.0), and mounted on glass cover slips in Fluorogel (Electron Microscopy Sciences, Hatfield, PA USA) suspended by a CultureWell chambered cover glass (Grace Biolabs Bend, OR USA), and imaged with an Olympus FV500 Confocal Laser Scanning Microscope using an Olympus Ix81 inverted microscope (Olympus, Center Valley, PA, USA). Series of Z-axis confocal optical images were collected by a technician with no knowledge of sample identities from three random sites of the forestomach tissue with a 60× oil lens using the dual excitation and emission mode (excitation laser lines: 488 nm and 543 nm, emission filters: 525 nm and 600 nm, respectively). In three Z-stacks per sample, bacteria cells stained with propidium iodide (the 600 nm red fluorescence) in each of the optical images were counted and pooled for the image analysis using a method described previously [44]. Using ImageJ [45], *L. reuteri* biofilm formation was quantified by determining the red-channel pixel area in images captured from three separate fields of view per individual sample (which results in a total area of 0.144 mm^2^ per mouse). The auto-fluorescence of the mouse forestomach tissue was captured as background (488 nm excitation and 525 nm emission). Dual-color (red-colored bacteria and green autofluorescence background) confocal images with extended depth of focus (overlapping all z-optical stacks) were used for presentations in figures 1–3. For 3D rendering, the fixed tissue was imaged using a Nikon A1 upright scanning confocal microscope (Nikon, Melville, New York) at 1 µM slices and rendered using the Nikon Analysis software.

### *In vitro* biofilm

*L. reuteri* 100-23 was grown in MRS supplemented with 1% maltose, 0.5% fructose and 0.1% sucrose (suMRS; pH 5.5) overnight and, after subculture (1% inoculum), for another 8 hours. 2.5 mL of this culture was injected into a disposable convertible flow cell with plastic (PET) cover slip (IBI Scientific, Peosta IA USA) which had been pre-conditioned with half-strength suMRS (pH 5.0; 37°C) as described previously [46]. Media flow from a reservoir of sterile half-strengh suMRS (pH 5.0; 37°C) was started 30 min after inoculation and maintained at a rate of 48 mL/h for 24 h (leading to six replacements of the chamber volume per hour). After 24 h, the flow chamber was carefully opened, and the biofilm was recovered in 3 ml growth media and immediately added to 7 ml RNAprotect. After 5 min incubation at room temperature, RNA was extracted as described below. Three individual *in vitro* biofilms were used to generate triplicate biological replicates. To compare the transcriptome of *L. reuteri* 100-23 cells grown in biofilms with that of planktonic cells, 100 mL batch cultures (three biological replicates) of prewarmed (37°C) suMRS (pH 5.5) were inoculated with 1% of an overnight culture of *L. reuteri* 100-23 grown in the same medium. Batch cultures were incubated for 4 h at 37°C to an OD_600_ of around 0.6. 50 mL were harvested by centrifugation (3 min for 3000× g) at 4°C, resuspended in 5 ml of 1 vol mMRS and 2 vol RNAprotect, and incubated for 10 min at room. Cells were recovered by centrifugation and subjected to RNA extraction. At harvest, the cultures were at pH 5 (+/−0.2), and therefore almost identical to the pH of the culture medium used for biofilm growth.

### RNA extraction and purification

RNA was extracted from *in vitro* biofilms and batch cultures (for transcriptome analysis) and from frozen forestomach samples and 8 h *in vitro* cultures (for qRT-PCR analysis). Bacterial cells from biofilm and batch cultures were collected by centrifugation and homogenized in 1 mL TRI Reagent (Molecular Research Center, Inc Cincinnati, OH USA) (Molecular Research Center, Inc., Cincinnati, OH USA). Cells were disrupted with three one-minute intervals in a Mini-Bead Beater (BioSpec Products, Inc. Bartlesville, OK USA) using zirconia/silica beads and cooling tubes on ice for one minute between intervals. Frozen forestomachs were added to 1 ml TRI Reagent and homogenized using the same conditions. Total RNA was extracted from these solutions according to the TRI Reagent instructions. Genomic DNA was removed using the TURBO DNA-free kit (Applied Biosystems/Ambion Austin, TX USA) followed by on-column DNase-treatment using the Qiagen RNeasy Kit (Qiagen Valencia, CA USA). DNase-treated RNA was quantified using the Nanodrop-1000 (NanoDrop Technologies, Wilmington, DE USA) and overall RNA integrity was determined in a RNase-free 1.2% agarose gel.

### Transcriptome comparison of cells grown in biofilm and planktonic cultures by microarray analysis

For the transcriptome work, the quality and concentration of RNA was determined using an Agilent 2100 Bioanalyzer (Agilent, Palo Alto, CA USA) and a NanoDrop ND-1000 Spectrophotomoter (ThermoScientific Wilmington, DE USA). Spotted microarrays containing probes for each of the annotated ORFs of *L. reuteri* 100-23 [16] were used for the experiment. Total RNA was directly labeled by reversed transcription using SuperScript II Reverse Transcriptase (Invitrogen, Carlsbad, CA) according to the manufacturer's instruction. The 20 µL reaction mix included 20 µg total RNA, random hexamers (200 ng/µL), 0.01 M dithiothreitol, 0.05 mM dATP, 0.05 mM dTTP, 0.05 mM dGTP and 0.02 mM dCTP, SUPERase (2 U/µL), 3.75 nM Cy3-dCTP dye or Cy5-dCTP (GE Healthcare UK limited, Little Chalfont Buckinghamshire, UK), and reverse transcriptase (30 U/µL). The reaction was incubated at 42°C for 2 h and terminated by adding 3 µL of 0.2 µM-filtered 0.5 M EDTA (final concentration 0.05 M) and an incubation for 2 min at RT. The RNA was removed by adding 3 µL 0.2 µM-filtered 1 M NaOH (final concentration 0.1 M) and incubating at 65°C for 30 min. The solution was neutralized by adding 3 µL 0.2 µm-filtered 1 M HCL. The labeling concentration was measured using a Nanodrop, and equal amounts of Cy3 and Cy5 labeled cDNAs were mixed together and purified using a QIAquick PCR purification kit according to the manufacturer's instruction (Qiagen Valencia, CA USA). 22 µL of LowTemp Hybridization buffer (ArrayIt Corporation, Sunnyvale, CA, USA) was used for elution. The final hybridization solution was prepared by mixing the 22.0 µL labeling mix, 3.5 µL Salmon sperm DNA (5 mg/mL) and 2.0 µL yeast tRNA (9.2 mg/mL). The hybridization was incubated at 43°C in dark overnight (approximately 16–20 h). The hybridized chips were washed using 1× SSC buffer plus 0.03% SDS, followed by 0.2× SSC, then 0.05× SSC for 5 min at room temperature sequentially with gentle agitation. Slides were immediately scanned with an Axon GenePix 4000 scanner (Axon, Union City, CA). Images were subsequently analyzed using Axon GenePix 4.0 software (Axon, Union City, CA). The experiment was performed in triplicate with biologically independent samples. The statistical analysis was carried out using R/Bioconductor and the LIMMA analysis package [47]. The complete data set of the gene expression analysis by microarray is presented in Table S3.

### Transcriptome comparison of cells grown in biofilm versus planktonic cultures using RNA sequencing (RNA-seq)

RNA from one sample of each condition (biofilm and batch culture) was subjected to the MICROBExpress Bacterial mRNA purification kit to reduce 16S and 23S rRNAs in the sample. The resulting RNA was subjected to standard Illumina library preparation and sequenced with an Illumina GAII sequencer, generating 16,004,489 (batch culture) and 14,005,687 (biofilm) reads of 50 bp length. Sequence reads were quality filtered resulting in 13,280,611 and 11,957,648 reads for the batch and biofilm culture, respectively. The reads were mapped to the *L. reuteri* 100-23 genome (NZ_AAPZ00000000.2) using Bowtie [48] while omitting reads that mapped to multiple locations or contained mismatches. This resulted in 822,571 (batch culture) and 693,758 (biofilm) reads that uniquely mapped to a library of ORFs constructed from the annotated 100-23 genome. The number of reads per ORF per condition was compared using GFOLD [49], which calculates a generalized fold change to identify differentially expressed genes. The complete dataset of the gene expression analysis by RNAseq is presented in Table S3.

### Quantitative Reverse Transcription PCR (qRT-PCR)

DNase-treated RNA isolated from forestomach tissues and 8 hr cultures was reverse transcribed using the Superscript VILO RT kit according to the manufacturer's instructions using the manufacturer's random primers (Invitrogen CA USA). Briefly, 20 µL reactions, containing approximately 1 µg of total RNA, of the Superscript VILO RT reaction were incubated for 10 min at 25°C, 60 min at 42°C and the reaction was terminated by heating to 85°C for 5 min. qRT-PCR was carried out on an Eppendorf Mastercycler Realplex2 machine (Eppendorf AG, Hamburg, Germany) using the Quanti-Fast SYBR Green PCR kit and primers designed with Primer3 [50] (Table S4). Primers were validated using serial ten-fold dilutions of pooled cDNA to determine specificity and efficiency. Tenfold dilutions of pooled cDNA were also included in each PCR reaction as efficiency controls. Efficiency controls were carried out in triplicate and experimental samples were performed in duplicate. For RT-PCR reactions, 12.5 µL of 2× Quantifast SYBR Green Mastermix (Qiagen Valencia, CA USA), 1 µL of ten-fold diluted cDNA, and 25 pMol of each primer were used per 25 µL reaction. A five-min denaturation step at 95°C was followed by 40 2-step cycles of 10 s at 95°C, then 30 s at 60°C. To confirm specificity of the PCR, products from each reaction were validated on an agarose gel and through inspection of their melting curves (denaturation step of 15 s at 95°C, an increase from 60°C–95°C over a 20 min period, and a final step of 15 s at 95°C). Gene transcripts were quantified relative to the glyceraldehyde-3-phosphate dehydrogenase housekeeping gene, whose expression did not differ between biofilm and batch culture growth (Table S1). Relative quantification of gene expression was performed using the method of Pfaffl [51] and compared using one-way ANOVA followed by Tukey's post-test.

### Gene inactivation

Insertional inactivation of target genes and *in vitro* characterization of mutant strains was carried out as described previously [30]. Growth experiments revealed that none of the mutants had any growth defects (data not shown).

### Proteomic analysis of cell-wall associated and secreted proteins

*L. reuteri* 100-23C wild-type and *secA2* mutant strains were subcultured overnight at 37°C in suMRS broth (pH 6.2) and mutant strains received erythromycin supplementation at 5 µg/mL. Cultures in 20 mL suMRS broth (pH 6.2) without antibiotic were subsequently inoculated ×1/100 and incubated at 37°C for 12 h. Cells were collected by centrifugation at 3000×*g* for 10 min at 4°C. Spent culture media samples (10 mL) were buffer-exchanged into TE1/1 (1 mM Tris-HCl, 1 mM EDTA, pH 8.0) and concentrated 35-fold by ultrafiltration through 3 kDa MWCO Ultra-4 spin filters (Amicon) at 4000×*g* and 4°C. Pelleted cells were washed with 5 mL ice-cold TES buffer (10 mM Tris-HCl, 1 mM EDTA, 25%, w/v, sucrose, pH 8.0) and re-centrifuged at 4°C for 2 min at 17000×*g*. Cell surface extracts were prepared by digesting whole cells in 2 mL TES buffer containing 6 mg/mL (577 kU/mL) lysozyme and 18 µg/mL (200 U/mL) mutanolysin for 3 h at 37°C. The treated cells were incubated on ice for 15 min and centrifuged at 4°C for 10 min at 2500×*g* and the supernatants containing released cell surface proteins removed carefully by pipette to avoid cellular contamination. Cell surface extracts were buffer-exchanged with TE1/1 buffer containing Complete Protease Inhibitor Cocktail and EDTA (Roche) and concentrated 15-fold by ultrafiltration through 10 kDa MWCO Ultra-0.5 spin filters (Amicon) at 14000×*g* and 4°C. Samples of concentrated spent media and cell surface extracts were electrophoresed through 4–12% Bis-Tris gradient gels (Novex Invitrogen) with MOPS-SDS buffer for 50 min at 200V constant voltage, followed by fixing and staining with Colloidal Blue (Novex Invitrogen). HiMark Unstained High Molecular Weight Protein Standard (Invitrogen) was electrophoresed for comparison. Individual protein bands or larger regions of each gel lane were excised from the gel, gel pieces were cut into ∼1 mm cubes and washed with 2×15 min incubations in 500 µl of 200 mM ammonium bicarbonate (ABC) in 50% (v/v) acetonitrile (ACN; Fisher) to equilibrate the gel to pH 8.0 and remove the stain, followed by a 10 min incubation with 500 µl ACN. Cysteine thiol side chains were reduced by incubation with 500 µl of 10 mM dithiothreitol in 50 mM ABC for 30 min at 60°C before being alkylated with 500 µl of 100 mM iodoacetamide in 50 mM ABC for 30 min at room temperature. The gel pieces were then washed with 2×15 min incubations in 500 µl of 200 mM ABC in 50% (v/v) ACN followed by 10 min in 500 µl ACN to dehydrate and shrink the gel pieces before air drying. Proteins were digested by the addition of 100 ng trypsin (modified porcine trypsin; Promega) in 10 µl of 10 mM ABC, or a mixture of 100 ng trypsin and 100 ng endoproteinase GluC (Roche) in 10 µl of 10 mM ABC before incubation overnight at 37°C. Following digestion, the samples were acidified by incubating with 10 µl of 1% (v/v) formic acid for 10 min. The digest solution was removed from the tube into an Eppendorf tube and the gel pieces were then washed with 20 µl of 50% ACN for 10 min to recover more digest peptides from the gel. The combined extracted digest samples were dried down at the low drying setting on a Speed Vac SC110 (Savant) fitted with a refrigerated condensation trap and a Vac V-500 (Buchi). Samples were stored frozen at −80°C prior to LC-MS/MS analysis in a nanoflow-HPLC system (nanoACQUITY: Waters) and a LTQ-Orbitrap mass spectrometer (Thermo). Peptides were trapped on line to a Symmetry C18 Trap (5 µm, 180 µm×20 mm) which was then switched in-line to a UPLC BEH C18 Column, (1.7 µm, 75 µm×250 mm) held at 45°C. Peptides were eluted by a gradient of 0–80% ACN in 0.1% formic acid over 50 min at a flow rate of 250 nl min^−1^. The mass spectrometer was operated in positive ion mode with a nano-spray source at a capillary temperature of 200°C. The Orbitrap was run with a resolution of 60,000 over the mass range m/z 300–2,000 and an MS target of 10^6^ and 1 s maximum scan time. The MS/MS was triggered by a minimal signal of 2000 with an Automatic Gain Control target of 30,000 ions and maximum scan time of 150 ms. For MS/MS events selection of 2+ and 3+ charge states selection were used. Dynamic exclusion was set to 1 count and 30 s exclusion time with an exclusion mass window of ±20 ppm. Proteins were identified by searching the Thermo RAW files converted to MASCOT generic format by Proteome Discover (Thermo) and proteins were identified by interrogating the *L. reuteri* 100-23 proteome database using the MASCOT v2.2.06 search engine (Matrix Science Ltd) [52]. MASCOT data were compared using Scaffold 4 v4.0.5 (Proteome Software, Inc.) with stringent filter settings of a protein threshold of 99.9% (minimum protein identity probability), a minimum number of peptides of 2 (minimum number of unique peptides per protein for identification) and a peptide threshold of 99.9% (minimum certainty of peptide identification for the minimum number of peptides set).

### Statistics

Statistical analyses were carried out using Graph Pad Prism 5 (GraphPad Software, Inc., California). Means and standard error of the mean are used. Comparisons were performed by ANOVA with Dunnett's multiple comparison test for biofilm formation, or with Tukey's post-test for gene expression comparisons. Significance of *p*<0.05 is denoted by a single asterisk (*), *p*<0.01 as two asterisks (**), and *p*<0.001 by three asterisks (***).

## Supporting Information

Figure S1 Genomic loci containing genes for the LytS/R and LrgA/B systems in *L. reuteri* and related bacteria with % amino acid identity.
(TIF)

Figure S2 Diagrammatic representation of LysM-domain proteins in *L. reuteri* and other bacteria.
(TIF)

Table S1 Gene expression as measured by Expression Microarrays and RNA-Seq .
(XLSX)

Table S2 Proteomics of cell wall and secreted proteins from *L. reuteri* 100-23C wild type and *secA2* mutant *in vitro* using Scaffold 4 analysis of MASCOT MS data: total spectrum count displayed for each protein.
(XLS)

Table S3 Microarray and RNASeq expression data for all genes of *L. reuteri* 100-23C presented in respective tabs. Genes in bold were inactivated by mutation for *in vivo* experiments. Microarrays were analyzed by LIMMA (see Methods), and the RNASeq data was analyzed by GFOLD (see Methods). Annotations for all gene and probe identifiers are also provided.
(XLSX)

Table S4 qRT-PCR primers used in this study.
(DOCX)

Video S1 3D rendered image of biofilm on rodent forestomach 48 hours after colonization.
(AVI)
