# Supplementary figures and images for: Molecular Characterization of Host-Specific Biofilm Formation in a Vertebrate Gut Symbiont
Source: PLoS Genet. 2013 Dec 26;9(12):e1004057. doi: 10.1371/journal.pgen.1004057 (PMC3873254; doi:10.1371/journal.pgen.1004057)

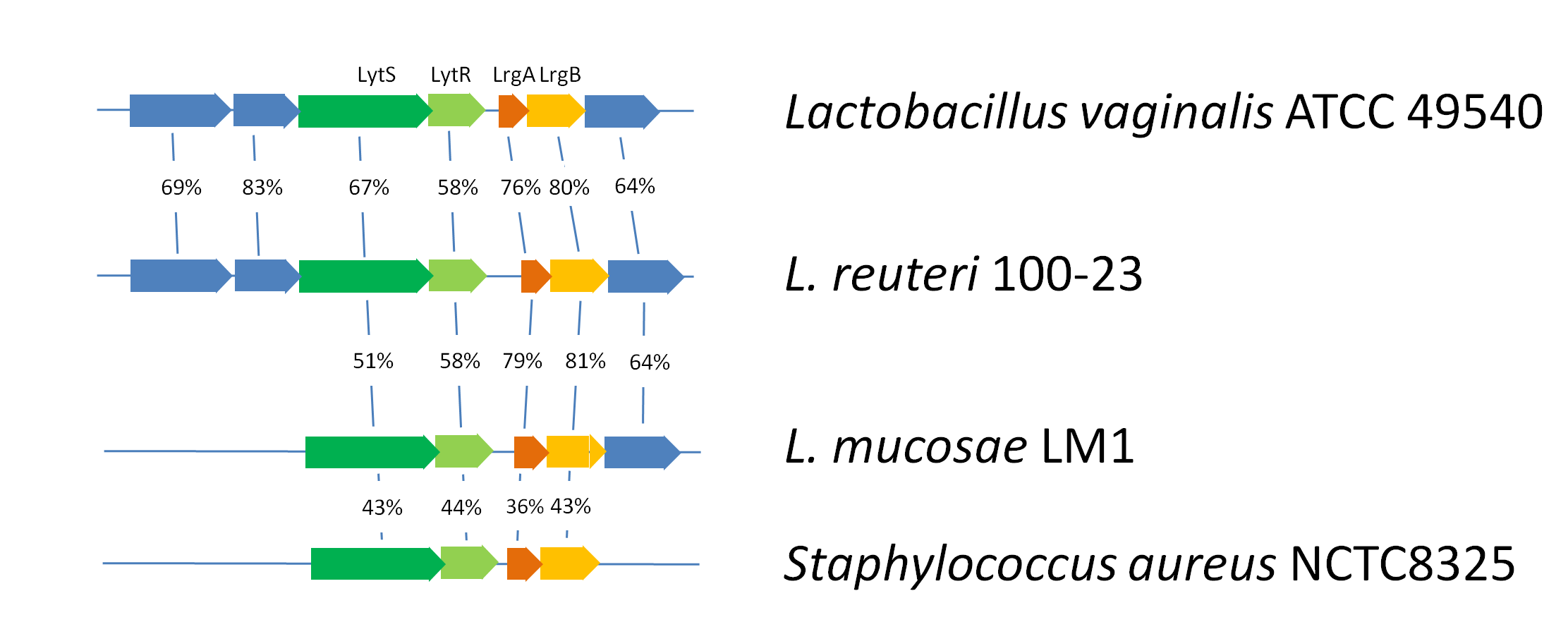

Supplement: Figure S1 — Genomic loci containing genes for the LytS/R and LrgA/B systems in L. reuteri and related bacteria with % amino acid identity. (TIF) [file pgen.1004057.s001.tif]

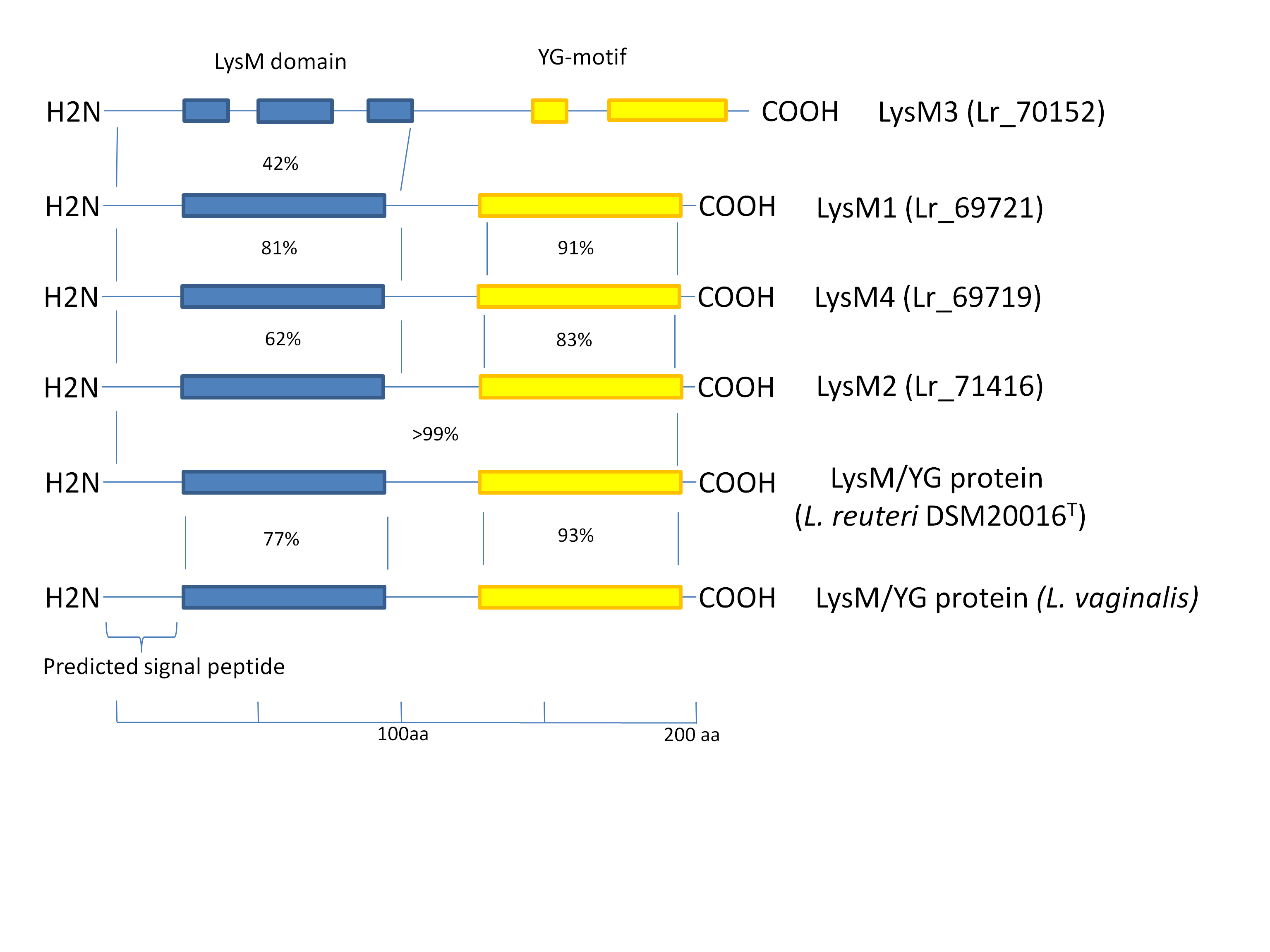

Supplement: Figure S2 — Diagrammatic representation of LysM-domain proteins in L. reuteri and other bacteria. (TIF) [file pgen.1004057.s002.tif]
