# Supplementary material for: Molecular Characterization of Host-Specific Biofilm Formation in a Vertebrate Gut Symbiont
Source: PLoS Genet. 2013 Dec 26;9(12):e1004057. doi: 10.1371/journal.pgen.1004057 (PMC3873254; doi:10.1371/journal.pgen.1004057)
Supplement: Table S4 — qRT-PCR primers used in this study. (DOCX) [file pgen.1004057.s006.docx]

| Gene | F/R | Oligonucleotide sequence (5' -> 3') |
| --- | --- | --- |
| Glyceraldehyde-3-phosphate dehydrogenase | F | CGGATTCACGAACTTAACACAA |
|  | R | CCTTACCATCAACAACAATAC |
| *lr69360* cystathionine γ-lyase | F | CGGAACTTTGGAGCAATGAT |
|  | R | GGTCCTCAATTCCCACTGAA |
| *lr70902* LPXTG-surface protein | F | ACACGGTCCAAGCTGAAGAT |
|  | R | AAATTCCACCGTTTCCATCA |
| *lr70580* lsp surface protein | F | ATACACCAGATTGGGCTTCG |
|  | R | CGCCCCAAGTTACAGTTTGT |
| *lr70892* secA2 transport protein | F | GCAACCTCACCTTTTGTGGT |
|  | R | CCCGTGTTTCGCTAACAAAT |
| *lr70114* urease α-subunit | F | AACTAACCCATATTGTAAGAACA |
|  | R | AGAAGTCATCATACTAAGGGCA |
| *lr71416* LysM2 | F | TGCTACTCTTGGTGCAGTGG |
|  | R | TTTCACCATCGCTCTTGATG |
| *lr70152* LysM3 | F | AATAACATGGCGATGCAACA |
|  | R | CGCTTCCTTCACGGTTTAAG |
| *lr69271* LrgA antiholin | F | GCCCATTTTAGTTCAAAT |
|  | R | TGGGACAAACAGAAAAGCAA |
| *lr69269* LytS putative regulator of cell autolysis | F | CAAATAACCAGTTGCATAACA |
|  | R | ACCCCTGCCACTAACCCTAC |
| *lr70532* ABC type bacteriocin transporter | F | TTTTCGCCTGCTAAGGGATA |
|  | R | CGAGGGAATAAACCGATCAA |
| *lr70430* two-component regulator | F | TCGAATAATTGGGATCGCTTA |
|  | R | TTTTGAGCCGTTGCTAATCC |
